# Association between Infant and Young Child Feeding (IYCF) Indicators and the Nutritional Status of Children (6–23 Months) in Northern Ghana

**DOI:** 10.3390/nu12092565

**Published:** 2020-08-24

**Authors:** Stephen Kofi Anin, Mahama Saaka, Florian Fischer, Alexander Kraemer

**Affiliations:** 1 School of Public Health, Bielefeld University, P.O. Box 100131, 33501 Bielefeld, Germany; florian.fischer@rwu.de (F.F.); alexander.kraemer@uni-bielefeld.de (A.K.); 2 Department of Industrial and Health Sciences, Faculty of Applied Sciences, Takoradi Technical University, P.O. Box 256, WS000 Takoradi, Ghana; 3 Department of Nutritional Sciences, School of Allied Health Sciences, University for Development Studies, P.O. Box TL 1883, NT000 Tamale, Ghana; mmsaaka@gmail.com; 4 Institute of Gerontological Health Services and Nursing Research, Ravensburg-Weingarten University of Applied Sciences, 88250 Weingarten, Germany

**Keywords:** stunting, wasting, underweight, malnutrition, undernutrition, complementary feeding practices

## Abstract

Although recommended infant and young child feeding (IYCF) practices have been found to be protective against undernutrition in some settings, there is no finality yet due to inconsistencies in the literature. A cross-sectional survey of 581 mother-child pairs was conducted in northern Ghana in June 2018. The association between IYCF indicators and child undernutrition (stunting and wasting) were assessed. The descriptive analysis showed that 66.4% of the children (6–23 months) were introduced to complementary feeding in a timely manner, 69.4% met the minimum meal frequency, and 38.9% met the minimum acceptable diet daily. The prevalence of stunting, wasting, underweight and overweight was 33.2%, 14.1%, 27% and 2.6%, respectively. From the multivariable binary logistic regression, child gender, child age group and source of power for lighting the household were significantly associated with wasting. Intake of iron-rich foods, child age group, and maternal height were significantly associated with stunting after adjusting for confounders. The prevalence of the compliance with IYCF indicators was relatively high. None of the individual IYCF indicators showed significant association with undernutrition, except intake of iron-rich foods for stunting. Nutrition-specific interventions targeted at improving IYCF practices, dietary diversification and intake of nutrient-rich meals, should be adopted and scaled up to address undernutrition in northern Ghana.

## 1. Introduction

The global burden of malnutrition remains unacceptably high, with about 150.8 million, 50.5 million and 38.3 million children under five years of age stunted (too short for their ages-height for age z-score (HAZ)/length for age z-score (LAZ)), wasted (too thin for their weights- weight for height/length z-score (WHZ/WLZ)) or overweight (too heavy for their heights/lengths—(WHZ/WLZ)) respectively [1,2]. In general, there has been a progressive decline in stunting (chronic malnutrition) globally during the Millennium Development Goals (MDG) era (2000–2015) and the current Sustainable Developments Goals (SDG) period (2016–2025), except in sub-Saharan Africa (SSA) and South East Asia (SEA) [1]. Despite declines in undernutrition globally, several low- and middle-income countries (LMICs) are likely to miss the target of a 40% reduction in stunting by 2025 set by the World Health Organisation (WHO) without substantial improvement in context-specific interventions [3].

Although Ghana is a mature and stable democracy, classified as a lower-middle-income country, it remains one of the 34 high-burden countries (mostly from SSA and SEA) that account for 90% of global stunting among children under five years of age [4]. In the Northern Region of Ghana, the estimated prevalence of stunting (chronic malnutrition) is 33.1%, compared to a national average of 19% [5]. A stunting prevalence of 30% or above is considered serious and thus qualifies to be given premium attention as a public health concern [2,6]. The consequences of undernutrition, in the form of protein energy malnutrition (PEM) and micronutrient deficiency malnutrition (MDM) among children during their first 1000-day period (from pregnancy through to the 24th month of childhood) include high levels of child mortality and morbidity, along with impaired physical, cognitive and socio-economic development in developing and emerging countries [7,8]. Undernutrition has been described as the underlying cause of 45% of deaths of children under five years of age [7].

The seminal works of the Lancet Maternal and Child Nutrition Series estimates that the proven nutrition-specific and evidence-based interventions (EBIs) taken together, if scaled up to 90% coverage, would reduce the prevalence of stunting and wasting in the 34 high-burden countries (where 90% of the world’s stunted children live) by about 20% and 60%, respectively [4]. Thus, interventions that seek to address the proximal determinants of chronic and acute undernutrition when prioritised and scaled up, have an appreciable chance of stemming the tide of, but not necessarily eliminating, child growth impairments. The WHO, UNICEF and many experts in maternal and child nutritional health, have recommended the suitability of IYCF indicators as highly cost-effective and pragmatic nutrition-specific parameters for monitoring the progress of food intake and guiding the development of appropriate interventions against child malnutrition in developing countries [4,9]. The core indicators include: timely introduction to complementary feeding (TICF), minimum meal frequency (MMF) for specific child ages, minimum dietary diversity (MDD), minimum acceptable diet (MAD), exclusive breastfeeding (EBF), early initiation of breastfeeding (EIBF), continued breastfeeding at one year (CBF@1) and adequate intake of micronutrient-rich foods (MRF), including Iron, Vitamin A, Zinc (Zn) and Iodine (I) [4,10]. Self-reported food intake data over a defined period, such as 24-h food recall (24HFR), remain widely and reliably used in analytical cross-sectional survey studies of malnutrition (under—and overnutrition) to validly measure infant and young child feeding (IYCF) indicators [10,11,12].

Therefore, the question arises as to whether IYCF indicators (breastfeeding and complementary feeding), which are postulated to be associated with chronic (stunting) and acute (wasting) child anthropometric growth indicators in LMICs, are viable monitoring parameters to identify populations at risk. The purpose of this study was to: (1) measure the prevalence of complementary feeding (CF)-related IYCF indicators, (2) identify context-specific factors associated with child nutritional status and (3) determine the association of these CF-related IYCF indicators with child nutritional status among children (6–23 months) in the Northern Region of Ghana, after adjusting for potential confounders.

## 2. Methods

### 2.1. Study Setting

A cross-sectional survey was conducted in June 2018 across the Northern Region of Ghana, which has the country’s highest prevalence rate of stunting (33%) according to the Ghana Demographic and Health Survey 2014 report [5]. February and March are usually the hottest months of the year in northern Ghana. There are two climatic seasons in the northern regions, the short rainy season (May–August) and the long dry season (September–April), which is characterised by dry Harmattan winds. The majority of the region’s inhabitants are engaged in subsistence agriculture, with a significant proportion involved in trading. The harvest season is usually from October to December, when there is usually an abundance of staple foods such as maize, sorghum, millet and yams.

### 2.2. Study Design and Population of Interest

A community-based, analytical, cross-sectional study design was used. Together with their children (6–23 months), 634 mothers of reproductive age (15–49 years) were selected using a two-stage cluster sampling technique from stunting-endemic districts in the 21 districts of the Northern Region of Ghana (Figure S1).

The study participants were drawn from 25 communities in five of the 10 districts in the Northern Region with a relatively high prevalence of stunting (≥30%) amongst children under five years of age. This was based on a Nutrition Surveillance Report (NSR) on the Northern Region conducted by the Ghana Health Service and the University for Development Studies in November 2013, and the Ghana Demographic and Health Survey (GHDS) 2014 report on the Northern Region [5]. There are several languages and dialects spoken in northern Ghana. However, the major languages spoken in the selected study districts are Dagbani (Dagombas), Gonja and Nanumba. The interviews were conducted in the local languages spoken by the participants, with the interviewers using an English-language questionnaire.

### 2.3. Sample Size

The sample size was calculated based on the standard formula for one-point sample estimation. The primary outcome variable used to estimate the sample size was the population proportion of malnutrition prevalence (stunting) rate, based on the 2014 Ghana Demographic and Health Survey (GDHS) report on the Northern Region [5]. The GDHS 2014 reported a 33.1% prevalence (survey from September–December 2014) of stunting in the Northern Region. With an estimated 20% of the total population of the Northern Region being children under five years of age and a 33.1% prevalence of chronic malnutrition [5], premised on 80% power and an absolute precision of 5% at the 95% confidence level, the sample size determined was 284. Assuming a correction factor of 2.0 (the design effect) for cluster sampling, the required minimum sample size was 568 [13]. Allowing for a 5% non-response rate to cover limiting circumstances such as missing values, implausible data, damage or loss of completed questionnaires and withdrawal from the interview or anthropometric measurement exercise, the overall sample size was adjusted to 600.

A two-stage cluster sampling procedure was used to select the study participants. In the first stage, five from the list of communities in each selected district were selected randomly using lottery (fishbowl) sampling while ensuring an adequate geographical spread to obtain 25 clusters (communities) in total, as recommended for community-based cluster sampling surveys [14]. The five communities (clusters) per district served as the primary sampling units (PSUs). The communities (clusters) selected were also guided by the Nutrition Surveillance 2014 report on the Northern Region of Ghana, triangulated with information from the 2014 Ghana Demographic and Health Survey report on the Northern Region and the 2010 Population Census updated master list of communities and districts provided by the Metropolitan Health Directorates in the Northern Region.

In the second stage, households with eligible mother–child pairs were selected using a systematic random sampling (SyRS) method, thus serving as the secondary sampling units (SSUs) of the participants. This stage of the sampling involved selecting the 24 households from each cluster or community required to meet the estimated sample size of 600 for the study. The eligible households (6- to 23-month-old child; 15- to 49-year-old, non-pregnant mother) were identified and serially enlisted with the assistance of community volunteers, who usually had prior knowledge of the household characteristics and thus served as community entry guides in the field. After determining the sampling interval by dividing the number of eligible households by 24, any sampling interval was randomly selected as a starting point for the first household to visit, after which the subsequent houses were selected by adding the sampling interval to the first number selected. This was continued until all 24 households required per community or cluster had been selected for the definitive study. Based on the consent of the selected household to participate in the survey, mother–child pairs were selected and engaged for interview. In households where more than one child qualified (6–23 months), a child aged 6–12 months was selected randomly since complementary feeding is recommended to begin after the fifth month and stunting is relatively less prevalent amongst much younger children in the study age group [9,14].

### 2.4. Data Collection

With an estimated working sample size of 600 from 25 clusters (communities), a minimum of 24 mother–child pairs were interviewed in each community during June 2018. The field research team (data enumerators, supervisors and data-entry clerks), made up of undergraduates in community nutrition, were recruited mostly from the Northern Region and provided with training prior to the pilot and definitive surveys. A final version of the quantitative survey instrument was produced using a composite instrument from the combination of a WHO interviewer-administered questionnaire used for assessing food intake in malnutrition studies and a Food and Agriculture Organisation (FAO) food-intake (food security) questionnaire after reviewing inputs from the training, qualitative study, pre-testing and piloting activities [9,15].

The interviewer-administered questionnaire was used to elicit food intake and other relevant data from eligible mother–child pairs over a 24-h recall period.

### 2.5. Dependent Variables of the Study

The nutritional status measures (LAZ for stunting, WLZ for wasting and WAZ for underweight) of the children were the dependent variables in this study (Figure S2). Anthropometric measurements were taken to determine the nutritional status of the children and mothers by measuring their heights/lengths and weights. The data collected were used to calculate the anthropometric indicators of underweight, stunting, wasting and overweight respectively: WAZ (weight-for-age), LAZ (length-for-age), WLZ (weight-for-length: standard deviation (SD) < −2) and WLZ (weight-for-length: SD > +2) for the children and BMI (body mass index) for the mothers using the WHO Anthro Software Version 3. The data was exported to SPSS version 25 software for further analysis. The Z scores were based on the 2006 WHO growth standards, expressed as standard deviation units from the median value for the WHO growth reference groups [16]. Implausible Z-scores (scores falling outside the WHO flags): WLZ −5 to 5; LAZ −6 to 6 and WAZ −6 to 5 were excluded from the data set. Children who fell below minus two standard deviations (−2 SD) from the median of the reference population for LAZ, WAZ or WLZ were classified as stunted, underweight or wasted, respectively. A child was classified as overweight when WLZ was above plus two standard deviations (+2 SD) from the median of the reference population [16].

The maternal and child weights were measured using a standard electronic scale sensitive to the nearest 100 g (Seca 890). The recumbent length of each child was measured in a supine position to the nearest 0.1 cm with a portable Infantometer. This supine measurement was taken by placing each child on his or her back between the slanting sides, ensuring that the child’s head was placed gently against the fixed top end. The child’s knees were held down gently by the anthropometrist while the movable foot-piece of the Infantometer was drawn up to touch the child’s feet at right angles to the legs [14,17]. Some children who could stand appropriately were measured standing. The WHO Anthro software automatically converts height to length for children aged less than 24 months [18]. For the mothers, height was measured in a standing position using a Seca microtoise stadiometer to the nearest 0.1 cm.

### 2.6. Independent Variables of the Study

Figure S2 shows the conceptual framework of factors or covariates associated with malnutrition (undernutrition), which was the basis for the selection and measurement of independent variables. The determinants of undernutrition were classified into proximal or immediate, intermediate or underlying and distal or basic factors which served as the independent variables of the study based on the United Nations Children’s Fund’s (UNICEF) hierarchical conceptual framework [4,8,19].

### 2.7. Proximal Factors

Nutrition-specific interventions and programmes are often targeted at the immediate determinants of undernutrition [7]. The proximal independent variables measured and explored in this study include the CF-related WHO/UNICEF core infant and young child feeding (IYCF) indicators (TICF, MMF, MDD, MAD and iron intake), ACF, vitamin A intake, frequency of illness or infection (morbidity), bilateral pitting oedema, age and sex of each child [4,8,9].

### 2.8. Intermediate Factors

Nutrition-sensitive interventions and programmes are often targeted at the underlying determinants of undernutrition [7,20]. The intermediate independent variables measured were: the child’s intake of cereal-only-based meals (porridge), household wealth index (HWI), type of fuel used for cooking, water, sanitation and hygiene (WASH) factors (type of sanitary facilities/toilet used, sources of drinking water and hygiene practices), child’s pre-school attendance, number of children under five years catered for, child’s birthweight, mother’s age, height, marital status, educational status and breastfeeding status, usage of insecticide-treated nets (ITN), religion, ethnicity, parental occupation, maternal BMI, place of delivery, maternal and child healthcare services (antenatal and post-natal care) [4,8].

### 2.9. Distal Determinants

The distal independent variables included district of residence, name of community, type of community (rural, peri-urban/semi-rural or urban) and community wealth index (CWI) [4,8].

### 2.10. Food Intake Assessment (Infant and Young Child Feeding (IYCF) Indicators)

The CF-related IYCF indicators (TICF, MMF, MDD, MAD and iron intake) were estimated from a self-reported 24-h food recall (24HFR). Each mother was asked to recall the number of times, in the 24 h immediately prior to the survey, her child had received any type of meal, snack or drink (complementary feeding), aside from breast milk, from the seven [7] food groups as classified by the WHO [10,14]. Timely introduction of complementary feeding (TICF) refers to the commencement of complementary feeding (introduction of solid, semi-solid and soft foods besides breast milk) at six months after birth. Minimum dietary diversity (MDD) refers to the proportion of children (6–23 months) who received foods from at least four out of the seven food groups. Dietary diversity score (DDS) refers to the score for the number of food groups out of the seven that each child has been fed from during the last 24 h, 7 being the highest and 0 being the lowest score. Minimum meal frequency (MMF) refers to the proportion of children (6–23 months) who received the minimum recommended number of complementary feeds during the previous 24 h, which depends on the child’s age, as classified by the WHO (CF ≥ 2 times for 6–8 months and ≥3 times for 9–23 months plus snacks for breastfeeding children, and ≥4 times in 24 h for non-breastfeeding children). Minimum acceptable diet (MAD) refers to the proportion of children who received both the MMF and MDD for their age category, as classified by the WHO. Appropriate complementary feeding (ACF) is an aggregate indicator additionally derived from TICF, MMF and MDD [13,21]. ACF, as a composite index in this study, refers to the proportion of children who received the MMF, MDD and commenced complementary feeding at six months after birth (TICF) as recommended by the WHO. Intake of micronutrient-rich foods (MRF) was also estimated for vitamin A and iron, using the 17 food groups as classified by the FAO [12,22]. Children who received meals including items from at least one of the three iron-rich food groups were classified as having had adequate iron intake. The iron-rich food groups included foods listed in group 8 (organ meat/offal), group 9 (flesh meats) and group 11 (fish and sea food) of the 17 groups of foods as categorized by FAO. Children were classified as having received none (0), low (1–3) or high (≥4) vitamin A intake out of the seven Vitamin A-rich food groups in the self-reported 24 h food recall.

### 2.11. Body Mass Index (BMI)

Body mass index (BMI) was calculated for the mothers as an indicator of nutritional status, reflecting chronic energy deficiency. BMI was calculated as weight (kg) divided by the individual’s height (in meters) squared. A BMI ≤ 18.5 was categorised as underweight or chronically energy deficient, BMI between 18.5 and 24.9 was classified as normal, BMI between 25 and 30 was classified as adequately nourished but overweight and BMI > 30 was classified as obese.

### 2.12. Bilateral Pitting Oedema

Oedema was assessed in the children by placing both thumbs on the upper section of the child’s feet and applying some pressure for about 2–3 s. Oedema was considered present when the skin depression on both feet remained after the pressure was released.

### 2.13. Household Wealth Index (HWI)

The socio-economic status (SES) of each mother–child dyad was determined using the first component from the principal component analysis (PCA) of the household ownership of certain durable assets, type of utilities and household space adequacy. The factor loadings were used to classify the participants into three levels of SES [23,24,25].

### 2.14. Data Entry and Analysis

Data from the interviewer-administered questionnaires (*n* = 634) was cleaned and analyzed using SPSS Version 25 software. WHO Anthro software version 3 was used for the Z-score computation from the anthropometric data. Cases with missing values or implausible Z-scores were deleted from the dataset. The cleaned data (*n* = 581) was tested for compliance with the key assumptions underlying multivariable binary logistic regression analysis. This was to ensure statistically valid inferences from the data. A descriptive analysis was performed in order to estimate the prevalence of nutritional status and IYCF indicators and was followed up with bivariate analyses to determine associations between the independent and dependent variables (stunting and wasting). An explanatory modelling approach was used to analyse the relationships between IYCF practices and undernutrition (stunting and wasting). Multivariable binary logistic regression analyses were performed to determine the associations between the IYCF indicators and undernutrition (stunting and wasting), accounting for the effects of other relevant factors and covariates. Bivariate analyses for all the categorical risk factors were performed using Chi-square (χ^2^) tests to measure association at *p* < 0.10 for the IYCF indicators and at *p* < 0.05 for the other factors or covariates [26,27,28]. Bivariate analyses for all the non-categorical independent variables were performed at *p* < 0.05 using Pearson’s correlation (r). To produce a parsimonious model, multicollinearity was assessed among the significant variables selected from the bivariate analyses, using a variance inflation factor (VIF) with a threshold of 3 for nominal variables and 10 for non-categorical variables. ACF and MAD violated the assumption of singularity because they are derived from a combination of TICF-MAD and MMF-MDD respectively. Furthermore, ACF and MAD showed multicollinearity with other IYCF indicators and were thus not included in the regression models. Intake of iron-rich foods was included in the regression model because of its biological and statistical significance (*p* < 0.10) [26,28]. The overall model performance and calibration were assessed using the Hosmer–Lemeshow goodness of fit (GOF) test and the Nagelkerke R^2^ [29]. The independent variables found to be significantly associated with each of the dependent variables in the bivariate analyses were used in the multivariable binary logistic regression modelling at *p* < 0.05. Adjusted odds ratios (AOR) and 95% confidence intervals (CI) were reported at *p* < 0.05 level of significance.

### 2.15. Ethical Clearance and Community Entry Protocols

Ethical clearance was secured from the Ghana Health Service Ethics Review Committee (GHS-ERC: 011/11/17) in Accra, Ghana, and the Ethics Committee of Bielefeld University (EUB 2018-083) in Bielefeld, Germany. After explaining the purpose and scope of the study to the participants in their local languages, written informed consent prior to enrolment was obtained from each mother (endorsed with a thumb print or signature on the consent forms), in addition to verbal consent from the household heads. Community entry protocols for the study area were followed accordingly.

## 3. Results

### 3.1. Characteristics of the Study Participants

The data for the 581 study participants from the five districts used for the analysis show that they were mostly (68.2%) from the Dagomba ethnic group. The mean (±SD) maternal and child ages were 29.31 ± 6.40 years and 13.25 ± 5.09 months respectively. Of the mothers, 90.2% were Muslims and a little over half (55.2%) were between 25 and 34 years of age. Almost all (97.7%) were married and most (94.3%) had basic education (completed only primary/junior high school), or no formal schooling at all. The majority (86.9%) of the mother–child pairs had no access to improved toilet facilities in their households. A little over half (52.2%) of the study participants had household access to improved sources of drinking water. A significant majority (91%) had access to electricity as a means of lighting their homes and powering electrical devices (Table 1 and Table 2, Table S1).

Using principal component analysis (PCA) of the ownership of some durable household assets, access to utilities and household space adequacy, 39.9% and 40.3% of the study participants were classified as being of low and medium-level socio-economic status (SES) respectively (Table 1 and Table 2, Table S1).

### 3.2. Nutritional Status of the Children by Age

The prevalence of stunting (LAZ < −2SD), wasting (WLZ < −2SD), underweight (WAZ < −2SD) and overweight (WLZ > +2SD) was 33.2%, 14.1%, 27% and 2.6%, respectively (Table 3). Of these, 79.3%, 65.9%, 70.7% and 60% respectively were aged 12–23 months.

The prevalence of the IYCF indicators estimated were TICF: 66.4%, MMF: 69.4%, MDD: 50.6%, MAD: 38.9%, ACF: 28.2%, intake of vitamin A-rich foods (63.2%) and intake of iron-rich foods (58.0%) (Table 4).

### 3.3. Bivariate Association between World Health Organisation (WHO) IYCF Indicators and Stunting

In the bivariate analysis, intake of Vitamin A-rich foods (Vitamin A), MDD and MAD were significantly associated with stunting at *p* < 0.05 while ACF and iron were significantly associated with stunting at *p* < 0.1 (Table 5). The covariates or factors found to be significantly associated (*p* < 0.05) with stunting include the district of residence, maternal age, religion, tribe, child age group, maternal height, number of postnatal care (PNC) visits, usage of insecticide-treated nets (ITN) and number of occupants per household (Table S2).

### 3.4. Bivariate Association between WHO IYCF Indicators and Wasting

None of the IYCF indicators was significantly associated with acute undernutrition (wasting) in the bivariate analysis at *p* < 0.10 (Table S3). The covariates or factors found to be significantly associated (*p* < 0.05) with wasting include religion, marital status, tribe, child gender, child age group, maternal BMI and the utility power source used for lighting households. However, child morbidity (any sickness in the last two weeks), frequency of diarrhoea during the last six months, child immunization status, postnatal visits to a healthcare facility and the frequency of cereal-only-based porridge consumption were associated with wasting at *p* < 0.10 (Table S4).

### 3.5. Multivariable Association between IYCF Indicators and Stunting (LAZ)

None of the IYCF indicators was significantly associated with stunting after adjusting for potential confounders except intake of iron-rich foods. Child age group and maternal height were significantly associated with stunting among the children studied in the Northern Region (Table 6). These predictors explained 27.8% (Nagelkerke R^2^ = 0.278) of the variance in the outcome of stunting. The Hosmer and Lemeshow GOF test for the final model was not statistically significant (χ^2^ = 5.282, df = 8, *p* = 0.727), which is indicative of model adequacy.

### 3.6. Multivariable Association between IYCF Indicators and Wasting (WLZ)

None of the IYCF indicators was significantly associated with wasting after adjusting for potential confounders. Child gender, child age group and source of power for lighting households were significant predictors (*p* < 0.05) of wasting among the children studied in the Northern Region (Table 7). These factors explained 18.5% (Nagelkerke R^2^ = 0.185) of the variance in the outcome of wasting among the children. The Hosmer and Lemeshow GOF test for the final model was not statistically significant (χ^2^ = 9.904, df = 8, *p* = 0.272), which is indicative of model adequacy.

Compared to male children, female children were about 0.42 times less likely to be wasted (AOR = 0.424, 95% CI: 0.248–0.725, *p* = 0.002). Compared to children aged 6–11 months, children aged 12–17 months were almost twice more likely to be wasted (AOR = 1.977, 95% CI: 1.048–3.730, *p* = 0.035). Compared to children from households with electricity to power lighting and other electrical devices, children who lived in households without electricity were 0.35 times less likely to be wasted (AOR = 0.353, 95% CI: 0.164–0.761, *p* = 0.008). Child age contributed most to the variance in wasting (Wald = 11.234, *p* < 0.004).

## 4. Discussion

This analytical cross-sectional study sought to measure the prevalence of complementary feeding (CF)-related WHO/UNICEF IYCF indicators and child nutritional status, to identify factors associated with child nutritional status and to determine the association of the IYCF indicators with child nutritional status among children (6–23 months) in the Northern Region of Ghana, after adjusting for potential confounders. Bivariate and multivariable statistical analysis of the associations between the CF-related IYCF indicators and the nutritional status of children in northern Ghana were thus examined.

### 4.1. Prevalence of IYCF Indicators among Children (6–23 Months) in Northern Ghana

One of the main findings of this study was that, generally, the prevalence of the CF-related IYCF indicators estimated in the Northern Region of Ghana were relatively higher compared to the national and northern regional findings of the GDHS 2014 [5]. The prevalence of stunting remained unchanged compared to the GDHS 2014 findings, but the levels of wasting and underweight showed increases, despite improvements in the prevalence of the CF-related WHO/UNICEF core IYCF indicators.

The IYCF indicators are nutrition-specific indicators recommended by the WHO, UNICEF and other maternal and child nutritional health experts as suitable, highly cost-effective, evidence-based and pragmatic parameters for monitoring and evaluating progress in child nutritional health in order to inform the development of appropriate interventions against the various forms of undernutrition [4,9]. The findings of this study showed some interesting similarities and disparities compared with the IYCF indicators reported in some recent studies conducted both in the Northern Region of Ghana and nationally. The Multiple Indicator Cluster Survey Six (MICS 6) survey, conducted in 2017–2018, reported a national prevalence of 79%, 41%, 23% and 12% respectively for TICF, MMF, MDD and MAD among children aged 6–23 months [30]. The GDHS 2014, conducted in 2013, reported a prevalence of 45.2%, 17.9%, 14.1%, 47.7% and 55.9% for MMF, MDD, MAD, iron and vitamin A, respectively, among children (6–59 months) in the Northern Region [5]. The indicators from our study were generally higher compared with the GDHS 2014 findings for the Northern Region. In a nutrition surveillance (NS) study, conducted in November 2013, of the three northern regions of Ghana, Saaka et al. [14] also reported a prevalence of 48.8%, 58.2%, 34.8%, 27.8% and 15.7% for TICF, MMF, MDD, MAD and ACF respectively among children aged 6–59 months. The general improvements in IYCF indicators reported from our study could possibly reflect the various nutritional intervention strategies implemented in northern Ghana over the period between November 2013 and June 2018, when these studies were conducted.

### 4.2. Nutritional Status of Children (6–23 Months) in the Northern Region of Ghana

The prevalence of undernutrition in the Northern Region generally has not improved compared to that found in recent studies. For instance, compared to the estimated prevalence in the GDHS 2014 of stunting (33.1%), wasting (6.3%) and underweight (20%) among children (6–59 months) in the Northern Region of Ghana, stunting has remained the same, wasting has increased by a factor of about two, and underweight has also increased, by almost one-third [5]. The Multiple Indicator Cluster Five (MICS 5) survey, conducted in 2011, reported 37.4%, 8.1%, 24.2% and 1.1% for stunting, wasting, underweight and overweight prevalence respectively in the Northern Region [31]. MICS 6, conducted in 2017–18, reported 29%, 9% and 1% for stunting, wasting and overweight among children (6–59 months) in the Northern Region [30]. Saaka et al. [14] reported a prevalence of 20.5%, 11.5% and 21.1% for stunting, wasting and underweight respectively in a survey of 44 districts in the northern regions of Ghana conducted in November 2013 among about 1.3 million children under the age of five years. The disparities could be attributed to the age ranges of the children surveyed in these studies or the periods of the surveys, among other reasons. A disaggregation of the prevalence rates for children under two years of age (6–24 months) from the GDHS 2014 and the MICS 5 and 6 studies would be optimal for comparison with this study. Nonetheless, chronic undernutrition (LAZ), seems intractable in the Northern Region of Ghana. The majority of the stunted, wasted, underweight and overweight children were aged 12–23 months (Table 3). This observation supports the hypothesis that the peak period for complementary feeding among growing children, with or without continued breastfeeding and comorbidities, is critical to optimal child growth [32].

Compared to the GDHS 2014, MICS 5 and MICS 6, the prevalence of wasting (WAZ) in the Northern Region has increased [5,30,31]. Acute undernutrition or wasting (WLZ), which reflects the effect of acute inadequate nourishment among children, is a more useful measure in emergency situations than stunting, which reflects an impairment of growth due to prolonged inadequate nutrient intake. The level of wasting estimated in this study (14.1%) is sufficient to be classified as serious (10–14%) based on the WHO cut-off values for public health significance [2,6]. This is probably due to the period of the survey (June), during which the onset of rain ushers in the planting season, which is known for its scarcity of food and increase in infectious diseases such as malaria.

Underweight (WAZ) and overweight (WLZ) status were both present in this study population, at 27% and 2.6% respectively. Falling within the high prevalence category according to the WHO public health significance cut-off (20–29%), the level of underweight is a phenomenon reflective of the overall nutritional health of children, which is usually premised on one or both of chronic growth impairment and acute growth impairment due to prolonged famine, acute hunger or frequent illness, among other causes [2,6]. Although the prevalence of overweight status among the children was relatively low, the emerging phenomenon of the double or multiple burdens of malnutrition in developing countries calls for efforts to stem the tide before it becomes too widespread in the Northern Region. However, generally, the prevalence of the IYCF indicators is relatively higher, this did not translate into a decrease in the prevalence of stunting or wasting status of the children in northern Ghana. Instead, the indicators of undernutrition were either similar to or worse than before. Appropriate, context-specific interventions are therefore needed in order to address this public health menace in northern Ghana.

### 4.3. Association between IYCF Indicators and Undernutrition (Stunting and Wasting)

Intake of iron-rich foods, child age group and maternal height were significant predictors (*p* < 0.05) of stunting among the children studied in the Northern Region. Child gender, child age and the source of power for lighting in households were significant predictors (*p* < 0.05) of wasting among the children. None of the IYCF indicators showed a significant association with stunting or wasting after adjusting for potential confounders in the models, except intake of iron-rich foods for stunting (Table 6 and Table 7). This apparent lack of significant correlation between the IYCF indicators and child nutritional status may also be due to insignificant variations in the child feeding practices of the study population generally. The study was conducted during the lean season and dietary diversity may be altered during this period because most rural households depend on their own production of subsistence crops, livestock and birds. However, some studies in SSA and SEA countries found strong relationships between some of the complementary feeding (CF)-related indicators and nutritional status, while others reported no significant associations [25,33,34,35]. The inconsistent findings about the relationship between the CF-related IYCF indicators and child anthropometric growth indicators (LAZ, WLZ and WAZ) may thus be context-specific and moderated by other factors or covariates [4,36,37,38].

Similar to this study, Saaka et al. [14], in their quest to ascertain the relationship between the IYCF indicators and nutritional status of children in northern Ghana, found that none of the core CF-related indicators was a significant predictor of stunting (LAZ), except TICF. Interestingly, in this same study by Saaka et al. [14], ACF, a composite food intake (CFI) metric estimated from TICF, MMF and MDD, was a significant predictor of wasting, but not stunting. Reinbott et al. posit that a composite child feeding index (CCFI), computed from a combination of the IYCF core indicators, is superior to the individual IYCF indicators in explaining stunting among children in DCs [25]. The predictive utility of variously computed CCFIs from the individual IYCF indicators should be further investigated to validate or disprove the hypothesis that CCFIs are more resilient metrics than the individual IYCF indicators for predicting and monitoring progress in child nutritional health status, as postulated [25,34,39,40]. Also, in consonance with the findings of this study, none of the individual IYCF indicators was associated with LAZ in recent studies conducted in Cambodia and southern Ethiopia [25,41]. The fact that the general improvement in the individual IYCF indicators in the Northern Region found in this study did not necessarily show a corresponding improvement in the nutritional status of the children, is suggestive of a weak association between IYCF indicators and undernutrition (stunting and wasting). The predictive utility of the IYCF indicators individually for stunting and wasting is thus limited. This buttresses the observation that, although a prolonged state of limited or inadequate food intake has been implicated as a major cause of stunting, some emerging evidence now suggests that stunting could continue to prevail even in food-secure households (nutritionally negative deviant children) or vice versa in food-insecure households (nutritionally positive deviant children). This suggests that, among other things, sub-optimal food utilisation, in synergy with other proximal and intermediate factors, may potentially play a significant role in children’s nutritional status [42,43,44].

Intake of foods rich in iron has been reported as a significant determinant of anaemia, but not often for stunting among children in DCs [45,46,47]. Kubuga et al., in a 12-week, community-based feeding trial study in northern Ghana, found the consumption of iron-rich native *Hibiscus sabdariffa* leaf meal to be significantly associated with anaemia in women of reproductive age (15–49 years) and had a protective effect against stunting among toddlers [48]. This observation may be of interest and relevance in developing dual-purpose nutritional intervention programmes aimed at addressing both stunting and anaemia.

The finding about age as a significant risk factor for stunting is consistent with similar nutritional studies in northern Ghana and other DCs [13,15,49]. Compared to children in the youngest age range (6–11 months), older children especially had a significantly higher risk of stunting. During this period, complementary feeding tends to be compromised, leading to adverse child anthropometric growth, while infants aged 6–11 months appear to benefit from the nutrient-rich and protective effect of breastfeeding. Maternal height has been reported as significantly associated with stunting by studies in similar settings [15,38]. The epigenetic influence of maternal height tends to underscore the role of genes and the environment on phenotypic expressions in children, even though the WHO child growth standards depict that all children follow similar optimal growth patterns, irrespective of their race, type of feeding, wealth or social status, among other factors, under similar environmental circumstances all around the world [16,50].

The covariates or risk factors that were significantly associated with wasting after adjusting for confounders were child gender, child age group and source of power for lighting the household (Table 7). Acute undernutrition (wasting) tends to be relatively more pronounced in male children, probably due to diarrhoeal diseases arising from the greater tendencies of male children to explore their environment as they begin to crawl, toddle and/or walk [14,15,51,52]. Females are also genetically more resilient to stressors than males in early life [53]. Child age group was associated with wasting, as reported in a similar study in northern Ghana [54]. Young children, upon commencement of complementary feeding, are more acutely vulnerable to diarrhoeal infections and acute food inadequacies than other children much younger or older. Besides being introduced to complementary meals, breastfeeding provides babies (much younger children) with the required nourishment and protection against infections, whilst the relatively older children can better ingest the often more nutritious household meals than children in the intermediate age-groups (6–11 and 12–17 months). More often than not, these family meals will be more nutritious than the often monotonous cereal based-only porridge fed to young children [54]. Households without access to electricity are likely to have limited capacity to safely and properly preserve food ingredients and unused food meant for their children, thus increasing children’s vulnerability to unsafe food intake.

### 4.4. Strengths and Limitations of the Study

Every pragmatic and rigorous effort was made to obtain high quality primary data, involving standard interviewer training and a pilot study. The sample size was large and representative enough (80% power) of children aged 6–23 months in the Northern Region, in order to satisfactorily address the research questions of interest to the study. However, being a cross-sectional study, causality cannot be inferred from the results. Due to the limited availability of similar studies conducted in northern Ghana for the age range in focus in this study (6–23 months), some comparisons of the IYCF indicators were not ideal. Some of the studies whose findings were compared with this study involved children (24–59 months) who were outside the age range of this study. Despite the widely accepted validity and reliability of data obtained from self-reported, interviewer-administered 24 HFR in nutritional epidemiological studies in DCs, recall bias arising from socially desirable responses and forgetfulness still poses a challenge and may thus have affected some of the findings. The independent variables and potential confounders explored in this study may not be exhaustive, and thus our understanding of the relationships between IYCF indicators and undernutrition in northern Ghana will be limited if they were not accounted for.

### 4.5. Recommended Public Health Interventions to Address Child Stunting and Wasting

Stunting is reflective of chronic undernutrition, and thus the risk factors identified in this study inform the suggested interventions accordingly. The evidence that consumption of iron-rich foods makes a difference to stunting gives an indication of the need for some micronutrients. Nutritional interventions that improve the intake of iron-rich foods could serve the dual purpose of reducing both child stunting and anaemia. Increasing dietary diversity is a potentially viable approach to reduce the burden of stunting among young children, given that dietary diversity scores are widely acceptable predictors of the adequacy of micronutrient intake [55,56,57].

While further prospective studies are needed to determine the effects of feeding practices on linear growth, food-based interventions (including diversifying food production, dietary supplementation, dietary diversification and biofortification) have great potential to ensure food and nutrition security, combat micronutrient deficiencies, improve dietary quality and raise levels of nutrition, especially among vulnerable groups, which include children under five years of age [58]. In addition, awareness-building and communication programmes promoting livelihood and social behaviour change that support the increased production and consumption of nutrient-rich foods (such as green leafy vegetables) are needed to bring about positive behavioural changes relating to food and nutrition.

Furthermore, interventions that address dietary diversity, such as unconditional cash transfers and the community-based promotion of improved infant and young child feeding practices, should be pursued, together with efforts to reduce the burden of infectious diseases, such as seasonal malaria chemoprevention. Practices that promote the consumption of blended staples with other locally available protein-rich food sources should be encouraged to improve the quality of child food intake.

Strong advocacy by local, national, and international stakeholders who are engaged in sustainable human capital development in developing and emerging nations is recommended in order to improve children’s diets during the first two years of life, as a priority area. The government of Ghana needs to ensure that its food and nutrition security policies are locally relevant and factually appropriate but aligned with internationally agreed-upon recommendations in order to protect, promote and support age-appropriate complementary feeding practices for infants and young children. The government and its development partners, including nutrition and health-related non-governmental organisations (NGOs), need to better coordinate efforts in order to implement multisectoral, large-scale, evidence-based programmes for the promotion and support of improved complementary feeding for children aged 6–23 months in the Northern Region of Ghana.

Because wasting is reflective of acute undernutrition, it requires situational interventions. Nutrition-sensitive interventions, such as child immunization and vaccination programmes and promotion of appropriate water, sanitation and hygiene (WASH) practices to reduce the burden of infectious diseases, policies and programmes targeted at improving agriculture and food security (the availability and/or supply of food, purchasing power and optimal utilization of limited food resources) in households, should be implemented and scaled up. These could more sustainably address the limitations inherent in the overreliance on food donation and micronutrient supplementation programmes in northern Ghana to address the undesirable trend of acute nutritional status.

Nutrition-sensitive interventions, such as behaviour change education (BCE) targeted at improving nutrition-specific IYCF practices, should be continually carried out. Feeding and caregiving aimed especially at male children to minimise child morbidity, and nutritional education targeted at dietary diversification to improve the intake of nutrient-rich meals, should be adopted and scaled up during periods of food shortages or famine. The timing of such interventions should coincide with the period of highest food insecurity and highest vulnerability to infectious diseases, which is usually at the onset of the major planting season (May–August). Additionally, nutrition-specific interventions such as the production and distribution of special nutrient-rich foods, ready-to use therapeutic foods (RUTFs) or formulated meals from local food ingredients and the prompt treatment of sick children in resource-poor settings such as northern Ghana could help address acute undernutrition.

The influence of child gender and maternal height on undernutrition remains poorly understood and warrants further research. Alongside the conventionally known and widely researched determinants of undernutrition in developing countries (DCs), other potentially novel risk factors of undernutrition should also be investigated to ascertain their possible associations with the nutritional health of children in northern Ghana and other similar settings in DCs [59,60]. An example is the effects of traditional cereal processing methods (TCPMs) such as soaking, germination, wet-milling, dry-milling, dehulling, storage and fermentation on the nutritive and non-nutritive constituents of cereal ingredients (flour and dough) used for complementary meal preparation. More research into the food utilization dimension of household food security (Figure S2) including some potentially unhelpful or unhealthful habitual food resource utilization practices (HFRUPs) such as some religious, traditional or indigenous food preparation methods may be very helpful [60]. The influence of cultural beliefs and practices relating to child feeding and caregiving as potential risk factors for undernutrition could also be explored. Mycotoxin exposure as a potential risk factor for child growth impairment has been under investigation in some DCs for the last decade and should also be researched further in the northern regions of Ghana [43,61,62,63,64].

## 5. Conclusions

The prevalence of the CF-related IYCF indicators was relatively higher than in the GDHS 2014 findings but did not necessarily translate into improved nutritional status of children in the Northern Region of Ghana. Child age group showed consistent associations with stunting and wasting, thus supporting the need for age-appropriate feeding interventions for growing children. None of the individual CF-related IYCF indicators showed a significant association with undernutrition (stunting and wasting), except the intake of iron-rich foods for stunting.

## Figures and Tables

**Table 1:** Maternal characteristics (*n* = 581).

Characteristics	Frequency	%
Age *		
15–24 years	136	23.4
25–34 years	321	55.2
35–49 years	124	21.3
**Marital Status**		
Unmarried	16	2.8
Married	565	97.2
**Maternal Height**		
160 cm and above	282	48.5
Below 160 cm	299	51.5
**Occupation**		
Trader/Vendor/Manual Labourer	166	28.6
Farmer	323	55.6
Vocational/Skilled Service Worker	48	8.3
Unemployed	44	7.6
**Number of Postnatal Care (PNC) Visits**		
Fewer than 4 times	106	8.2
At least 4 times	475	81.8
**Number of Antenatal Care (ANC) Visits**		
Fewer than 4 times	34	5.9
At least 4 times	547	94.1
**Currently Breastfeeding**		
Yes	560	96.4
No	21	3.6

* Mean maternal age (±standard deviation (SD)) was 29.31 ± 6.40 years.

**Table 2:** Child Characteristics (*n* = 581).

Characteristics	Frequency	%
Age *		
6–11 months	242	41.7
12–17 months	185	31.8
18–23 months	154	26.5
**Gender**		
Male	301	51.8
Female	280	48.2
**Immunisation Status**		
Up to date	373	64.2
Not up to date	208	35.8
**Place of Birth**		
Family Home/Residence	225	38.7
Community-Based Health Planning and Services (CHPS) Compound/Traditional Maternity Home	45	7.7
Clinic/Health Centre	153	26.3
Hospital	158	27.2
**Usage of Insecticide-Treated Net (ITN)**		
Yes	518	89.2
No	63	10.8
**Recent Illness/Morbidity (within the two weeks immediately prior to the survey)**		
Yes	215	37.0
No	366	63.0
**Child’s Birth Weight (** ***n*** ** = 274)**		
Less than 2.5 kg	246	89.8
More than 2.5 kg	28	10.2
**Frequency of Feeding Child with Cereal-based-only Porridge**		
Always	137	23.6
Very often	129	22.2
Occasionally	187	32.2
No	128	22.0

* Mean child age was 13.25 ± 5.09 months.

**Table 3:** Nutritional status of children by age (*n* = 581).

Nutritional Status		Child Age						Total	
		6–8 Months		9–11 Months		12–23 Months			
		*n*	%	*n*	%	*n*	%	*N*	%
**Stunting (LAZ)**	No	120	30.9	82	21.1	186	48.0	388	66.8
	Yes	21	10.9	19	9.8	153	79.3	193	33.2
**Wasting (WLZ)**	No	126	25.3	88	17.6	285	57.1	499	85.9
	Yes	15	18.3	13	15.9	54	65.9	82	14.1 *
**Underweight (WAZ)**	No	116	27.4	80	18.9	228	53.8	424	73.0
	Yes	25	15.9	21	13.4	111	70.7	157	27.0 *
**Overweight (WLZ)**	No	135	23.9	101	17.8	330	58.3	566	97.4
	Yes	6	40.0	0	0.0	9	60.0	15	2.6 *

* Prevalence of Nutritional Status Indicator. Prevalence of infant and young child feeding (IYCF) Indicators among children (6–23 months) in Northern Ghana. Row % of cross tabulation are presented in the table, except for Total % of *n* in the last column.

**Table 4:** Prevalence of IYCF indicators by child age (*n* = 581).

IYFC Indicators		Child Age						Total (IYCF)	
		6–11 Months		12–17 Months		18–23 Months			
		*n*	%	*n*	%	*N*	%	*N*	%
**TICF: Timely Introduction to Complementary Feeding**	No	75	38.5	64	32.8	56	28.7	195	33.6
	Yes	167	43.3	121	31.3	98	25.4	386	66.4 *
**MMF: Minimum Meal Frequency**	No	94	52.8	48	27.0	36	20.2	178	30.6
	Yes	148	36.7	137	34.0	118	29.3	403	69.4 *
**MDD: Minimum Dietary Diversity**	No	177	61.7	61	21.3	49	17.1	287	49.4
	Yes	65	22.1	124	42.2	105	35.7	294	50.6 *
**MAD: Minimum Acceptable Diet**	No	193	54.4	84	23.7	78	22.0	355	61.1
	Yes	49	21.7	101	44.7	76	33.6	226	38.9 *
**ACF: Appropriate Complementary Feeding**	No	200	48.0	110	26.4	107	25.7	417	71.8
	Yes	42	25.6	75	45.7	47	28.7	164	28.2 *
**Intake of Iron-rich Foods**	No	155	72.4	31	14.5	28	13.1	214	36.8
	Yes	87	23.7	154	42.0	126	34.3	367	63.2 *
**Intake of Vitamin A-rich Foods**	No	145	59.4	59	24.2	40	16.4	244	42.0
	Low	93	28.6	126	38.8	106	32.6	325	55.9 *
	High	4	33.3	0	0.0	8	66.7	12	2.1 *

* Prevalence of complementary feeding (CF)-related IYCF indicator. Row % of cross tabulation are presented in the table, except for Total % of *n* in the last column.

**Table 5:** Bivariate analysis of the association between IYCF indicators and stunting (*n* = 581).

IYCF Indicators		Nutritional Status (*n*) %		Total (*N*) %	Test Statistic	
		Normal	Stunted			
**TICF**	No	(128) 65.6	(67) 34.4	(195) 100.0	χ^2^ = 0.172	* p* = 0.678
	Yes	(260) 67.4	(126) 32.6	(386) 100.0		
**MMF**	No	(124) 69.7	(54) 30.3	(178) 100.0	χ^2^ = 0.960	* p* = 0.327
	Yes	(264) 65.5	(139) 34.5	(403) 100.0		
**MDD**	˂4 foods	(212) 73.9	(75) 26.1	(287) 100.0	χ^2^ = 12.838	* p* < 0.001 *
	≥4 foods	(176) 59.9	(118) 40.1	(294) 100.0		
**MAD**	No	(251) 70.7	(104) 29.3	(355) 100.0	χ^2^ = 6.331	* p* = 0.012 *
	Yes	(137) 60.6	(89) 39.4	(226) 100.0		
**ACF**	No	(287) 68.8	(130) 31.2	(417) 100.0	χ^2^ = 2.781	* p* = 0.095 **
	Yes	(101) 61.6	(63) 38.4	(164) 100.0		
**Iron**	No	(153) 71.5	(61) 28.5	(214) 100.0	χ^2^ = 3.394	* p* = 0.065 **
	Yes	(235) 64.0	(132) 36.0	(367) 100.0		
**Vitamin A**	No intake	(183) 75.0	(61) 25.0	(244) 100.0	χ^2^ = 14.868	* p* = 0.001 *
	Low intake	(200) 61.5	(125) 38.5	(325) 100.0		
	High intake	(5) 41.7	(7) 58.3	(12) 100.0		

Significant at * *p* ˂ 0.05 and ** *p* ˂ 0.10; *n* = 581.

**Table 6:** Multivariable binary logistic regression analysis of predictors of stunting.

						95% CI	
IYCF Indicators	B	SE	Wald	Sig.	AOR	Lower	Upper
**Intake of Iron-rich foods (Yes)**	−0.735	0.328	5.028	0.025 *	0.479	0.252	0.912
**Child’s Age (6–11 Months)**			53.576	<0.001 *			
**Child’s Age (12–17 Months)**	1.481	0.285	27.078	<0.001 *	4.399	2.518	7.686
**Child’s Age (18–23 Months)**	2.158	0.296	53.169	<0.001 *	8.656	4.846	15.462
**Mother’s Height (Below 160cm)**	0.535	0.203	6.926	0.008 *	1.708	1.146	2.545
**Constant**	−2.507	0.766	10.701	<0.001	0.082		

* Significance at *p* < 0.05; AOR: Adjusted Odds Ratio; B: Beta value; SE: Standard error; CI: C Confidence interval.

**Table 7:** Multivariable binary logistic regression analysis of predictors of wasting.

IYCF Indicators						95% CI	
	B	SE	Wald	Sig.	AOR	Lower	Upper
**Child’s Gender (Female)**	−0.858	0.274	9.800	0.002 *	0.424	0.248	0.725
**Child’s Age (6–11 Months)**			11.234	0.004 *			
**Child’s Age (12–17 Months)**	0.681	0.324	4.425	0.035	1.977	1.048	3.730
**Child’s Age (18–23 Months)**	−0.473	0.401	1.391	0.238	0.623	0.284	1.367
**Power Source for Light** **(No Electricity)**	−1.041	0.391	7.070	0.008 *	0.353	0.164	0.761
**Constant**	0.591	0.946	0.390	0.532	1.805		

* Significance at *p* ˂ 0.05; AOR: Adjusted Odds Ratio; B: Beta value; SE: Standard error; CI: Confidence interval.

## Supplementary Materials

The following are available online at https://www.mdpi.com/2072-6643/12/9/2565/s1: Figure S1: Illustrative Map of the Northern Region of Ghana (www.ghanaweb), Figure S2. Conceptual Framework for the Determinants of Malnutrition (Undernutrition) in northern Ghana (Adopted and modified from Bhutta et al., 2013, Fanzo, 2012 and Muller and Krawinkel, 2005), Table S1: Socio-demographic and Household Characteristics *n* = 581, Table S2: Bivariate analysis of the association between factors and stunting (LAZ), Table S3: Bivariate analysis of the association between WHO IYCF indicators and Wasting, Table S4: Bivariate analysis of the association between factors and wasting.

## References

[1] Development Initiatives (2018). 2018 Global Nutrition Report Shining a Light to Spur Action on Nutrition, Bristol.

[2] United Nations Children’s Fund (UNICEF), World Health Organization, International Bank for Reconstruction and Development/The World Bank (2019). Levels and Trends in Child Malnutrition: Key Findings of the 2019 Edition of the Joint Child Malnutrition Estimates.

[3] Kinyoki D.K., Osgood-Zimmerman A.E., Pickering B.V., Local Burden of Disease Child Growth Failure Collaborators (2020). Mapping child growth failure across low-and middle-income countries. Nature.

[4] Bhutta Z., Das J.K., Rizvi A., Gaffey M.F., Walker N., Horton S., Webb P., Lartey A., Black R.E. (2013). Evidence-based interventions for improvement of maternal and child nutrition: What can be done and at what cost?. Lancet.

[5] Ghana Statistical Service (GSS), Ghana Health Service (GHS), ICF International (2015). Ghana Demographic and Health Survey 2014.

[6] De Onis M., Borghi E., Arimond M., Webb P., Croft T., Saha K., De-Regil L.M., Thuita F., Heidkamp R., Krasevec J. (2018). Prevalence thresholds for wasting, overweight and stunting in children under 5 years. Public Health Nutr..

[7] Black R.E., Victora C.G., Walker S.P., Bhutta Z., Christian P., De Onis M., Ezzati M., Grantham-McGregor S., Katz J., Martorell R. (2013). Maternal and child undernutrition and overweight in low-income and middle-income countries. Lancet.

[8] Fanzo J. (2012). The Nutritional Challenge in Sub-Saharan Africa.

[9] WHO, UNICEF, IFPRI, UCDavis, FANTA, AED, USAID (2008). Indicators for Assessing Infants and Young Child Feeding Practices: Part 1—Definitions.

[10] WHO, UNICEF, IFPRI, UCDavis, FANTA, AED, USAID (2010). Indicators for Assessing Infants and Young Child Feeding Practices. Part 2: Measurement.

[11] Brouwer-Brolsma E.M., Brennan L., Drevon C.A., Van Kranen H., Manach C., Dragsted L.O., Roche H., Andres-Lacueva C., Bakker S.J.L., Bouwman J. (2017). Combining traditional dietary assessment methods with novel metabolomics techniques: Present efforts by the Food Biomarker Alliance. Proc. Nutr. Soc..

[12] FAO (2018). Dietary Assessment: A Resource Guide to Method Selection and Application in Low Resource Settings.

[13] Saaka M., Larbi A., Mutaru S., Hoeschle-Zeledon I. (2016). Magnitude and factors associated with appropriate complementary feeding among children 6–23 months in Northern Ghana. BMC Nutr..

[14] Saaka M., Wemakor A., Abizari A.-R., Aryee P.A. (2015). How well do WHO complementary feeding indicators relate to nutritional status of children aged 6-23 months in rural Northern Ghana?. BMC Public Health.

[15] Ali Z., Saaka M., Adams A.-G., Kamwininaang S.K., Abizari A.-R. (2017). The effect of maternal and child factors on stunting, wasting and underweight among preschool children in Northern Ghana. BMC Nutr..

[16] Onis M. (2007). WHO Child Growth Standards based on length/height, weight and age. Acta Paediatr..

[17] De Onis M., Onyango A.W., Broeck J.V.D., Chumlea W.C., Martorell R. (2004). Measurement and standardization protocols for anthropometry used in the construction of a new international growth reference. Food Nutr. Bull..

[18] WHO (2009). WHO AnthroPlus for Personal Computers Manual: Software for Assessing Growth of the World’s Children and Adolescents.

[19] Müller O., Krawinkel M. (2005). Malnutrition and health in developing countries. Can. Med Assoc. J..

[20] Ruel M.T., Alderman H. (2013). Nutrition-sensitive interventions and programmes: How can they help to accelerate progress in improving maternal and child nutrition?. Lancet.

[21] Kassa T., Meshesha B., Haji Y., Ebrahim J. (2016). Appropriate complementary feeding practices and associated factors among mothers of children age 6–23 months in Southern Ethiopia, 2015. BMC Pediatr..

[22] FAO IaW (2013). The State of Food Insecurity in the World 2013: The Multiple Dimensions of Food Security.

[23] Doku D., Koivusilta L., Rimpelä A. (2009). Indicators for measuring material affluence of adolescents in health inequality research in developing countries. Child Indic. Res..

[24] Filmer D., Pritchett L.H. (2001). Estimating wealth effects without expenditure data-or tears: An application to educational enrollments in states of India. Demography.

[25] Reinbott A., Kuchenbecker J., Herrmann J., Jordan I., Muehlhoff E., Kevanna O., Krawinkel M. (2014). A child feeding index is superior to WHO IYCF indicators in explaining length-for-age Z-scores of young children in rural Cambodia. Paediatr. Int. Child Health.

[26] Ughade S. (2013). Statistical modeling in epidemiologic research: Some basic concepts. Clin. Epidemiol. Glob. Health.

[27] Bursac Z., Gauss C.H., Williams D.K., Hosmer D.W. (2008). Purposeful selection of variables in logistic regression. Source Code Boil. Med..

[28] Zhang Z. (2016). Model building strategy for logistic regression: Purposeful selection. Ann. Transl. Med..

[29] Tabachnick G.B., Fidell S.L. (2007). Using Multivariate Statistics.

[30] Ghana Statistical Service (2018). Multiple Indicator Cluster Survey.

[31] Ghana Statistical Service (2011). Ghana Multiple Indicator Cluster Survey (MICS) with an Enhanced Malaria Module and Biomarker 2011.

[32] Lassi Z.S., Das J.K., Zahid G., Imdad A., Bhutta Z. (2013). Impact of education and provision of complementary feeding on growth and morbidity in children less than 2 years of age in developing countries: A systematic review. BMC Public Health.

[33] Marriott B.P., White A., Hadden L., Davies J.C., Wallingford J.C. (2011). World Health Organization (WHO) infant and young child feeding indicators: Associations with growth measures in 14 low-income countries. Matern. Child Nutr..

[34] Bork K., Cames C., Barigou S., Cournil A., Diallo A. (2012). A summary index of feeding practices is positively associated with height-for-age, but only marginally with linear growth, in rural Senegalese infants and toddlers. J. Nutr..

[35] Jones A.D., Ickes S.B., Smith L.E., Mbuya M.N.N., Chasekwa B., Heidkamp R.A., Menon P., Zongrone A.A., Stoltzfus R.J. (2014). World Health Organization infant and young child feeding indicators and their associations with child anthropometry: A synthesis of recent findings. Matern. Child Nutr..

[36] Vossenaar M., Knight F., Tumilowicz A., Hotz C., Chege P., Ferguson E.L. (2016). Context-specific complementary feeding recommendations developed using Optifood could improve the diets of breast-fed infants and young children from diverse livelihood groups in northern Kenya. Public Health Nutr..

[37] Stewart C.P., Iannotti L., Dewey K.G., Michaelsen K.F., Onyango A.W. (2013). Contextualising complementary feeding in a broader framework for stunting prevention. Matern. Child Nutr..

[38] Beal T., Tumilowicz A., Sutrisna A., Izwardy D., Neufeld L.M. (2018). A review of child stunting determinants in Indonesia. Matern. Child Nutr..

[39] Lohia N., Udipi S. (2014). Infant and child feeding index reflects feeding practices, nutritional status of urban slum children. BMC Pediatr..

[40] Chaudhary S.R., Govil S., Lala M.K., Yagnik H.B. (2018). Infant and Young Child Feeding Index and its association with nutritional status: A cross-sectional study of urban slums of Ahmedabad. J. Fam. Community Med..

[41] Tessema M., Belachew T., Ersino G. (2013). Feeding patterns and stunting during early childhood in rural communities of Sidama, South Ethiopia. Pan Afr. Med J..

[42] Semba R.D., Shardell M., Ashour F.A.S., Moaddel R., Trehan I., Maleta K., Ordiz M.I., Kraemer K., Khadeer M.A., Ferrucci L. (2016). Child stunting is associated with low circulating essential amino acids. EBioMedicine.

[43] Chen C., Riley R.T., Wu F. (2018). Dietary fumonisin and growth impairment in children and animals: A review. Compr. Rev. Food Sci. Food Saf..

[44] Saaka M., Mutaru S. (2014). Factors contributing to positive deviance in the growth of children in rural northern Ghana. Int. J. Child Health Nutr..

[45] Mya K.S., Kyaw A.T., Tun T. (2019). Feeding practices and nutritional status of children age 6–23 months in Myanmar: A secondary analysis of the 2015–2016 Demographic and Health Survey. PLoS ONE.

[46] Mohammed S.H., Larijani B., Esmaillzadeh A. (2019). Concurrent anemia and stunting in young children: Prevalence, dietary and non-dietary associated factors. Nutr. J..

[47] Sachdev H., Gera T., Nestel P. (2006). Effect of iron supplementation on physical growth in children: Systematic review of randomised controlled trials. Public Health Nutr..

[48] Kubuga C.K., Hong H.G., Song W.O. (2019). *Hibiscus sabdariffa* meal improves iron status of childbearing age women and prevents stunting in their toddlers in northern Ghana. Nutrients.

[49] Tariq J., Sajjad A., Zakar R., Zakar M.Z., Fischer F. (2018). Factors associated with undernutrition in children under the age of two years: Secondary data analysis based on the Pakistan Demographic and Health Survey 2012–2013. Nutrients.

[50] Aguayo V.M., Nair R., Badgaiyan N., Krishna V. (2016). Determinants of stunting and poor linear growth in children under 2 years of age in India: An in-depth analysis of Maharashtra’s comprehensive nutrition survey. Matern. Child Nutr..

[51] Glover-Amengor M., Agbemafle I., Hagan L.L., Mboom F.P., Gamor G., Larbi A., Hoeschle-Zeledon I. (2016). Nutritional status of children 0–59 months in selected intervention communities in northern Ghana from the Africa RISING project in 2012. Arch. Public Health.

[52] Sahn D., Stifel D. (2002). Parental preferences for nutrition of boys and girls: Evidence from Africa. J. Dev. Stud..

[53] Wells J.C. (2000). Natural selection and sex differences in morbidity and mortality in early life. J. Theor. Boil..

[54] Sienso G., Lyford C. (2018). Assessing the factors affecting malnutrition in northern Ghana. J. Nutr. Disord. Ther..

[55] Zhao W., Yu K., Tan S., Zheng Y., Zhao A., Wang P., Zhang Y. (2017). Dietary diversity scores: An indicator of micronutrient inadequacy instead of obesity for Chinese children. BMC Public Health.

[56] Kennedy G., Pedro M.R., Seghieri C., Nantel G., Brouwer I. (2007). Dietary diversity score is a useful indicator of micronutrient intake in non-breast-feeding Filipino children. J. Nutr..

[57] Mallard S.R., Houghton L.A., Filteau S., Chisenga M., Siame J., Kasonka L., Mullen A., Gibson R.S. (2016). Micronutrient adequacy and dietary diversity exert positive and distinct effects on linear growth in urban Zambian infants. J. Nutr..

[58] Brian T., Amoroso L. (2014). Improving Diets and Nutrition: Food-based Approaches.

[59] Weerasooriya D.K., Bean S., Nugusu Y., Ioerger B.P., Tesso T. (2018). The effect of genotype and traditional food processing methods on in-vitro protein digestibility and micronutrient profile of sorghum cooked products. PLoS ONE.

[60] Hotz C., Gibson R.S. (2007). Traditional food-processing and preparation practices to enhance the bioavailability of micronutrients in plant-based diets. J. Nutr..

[61] Wild C.P., Gong Y.Y. (2009). Mycotoxins and human disease: A largely ignored global health issue. Carcinogenesis.

[62] Shirima C.P., Kimanya M.E., Kinabo J.L., Routledge M.N., Srey C., Wild C.P., Gong Y.Y. (2013). Dietary exposure to aflatoxin and fumonisin among Tanzanian children as determined using biomarkers of exposure. Mol. Nutr. Food Res..

[63] Wu F., Groopman J.D., Pestka J.J. (2014). Public health impacts of foodborne mycotoxins. Annu. Rev. Food Sci. Technol..

[64] Gong Y.Y., Watson S., Routledge M.N. (2016). Aflatoxin exposure and associated human health effects, a review of epidemiological studies. Food Saf..

