# Supplementary material for: Association between Infant and Young Child Feeding (IYCF) Indicators and the Nutritional Status of Children (6–23 Months) in Northern Ghana
Source: Nutrients. 2020 Aug 24;12(9):2565. doi: 10.3390/nu12092565 (PMC7551146; doi:10.3390/nu12092565)
Supplement: Supplementary file 1 [file nutrients-12-02565-s001.pdf]

Supplementary Materials

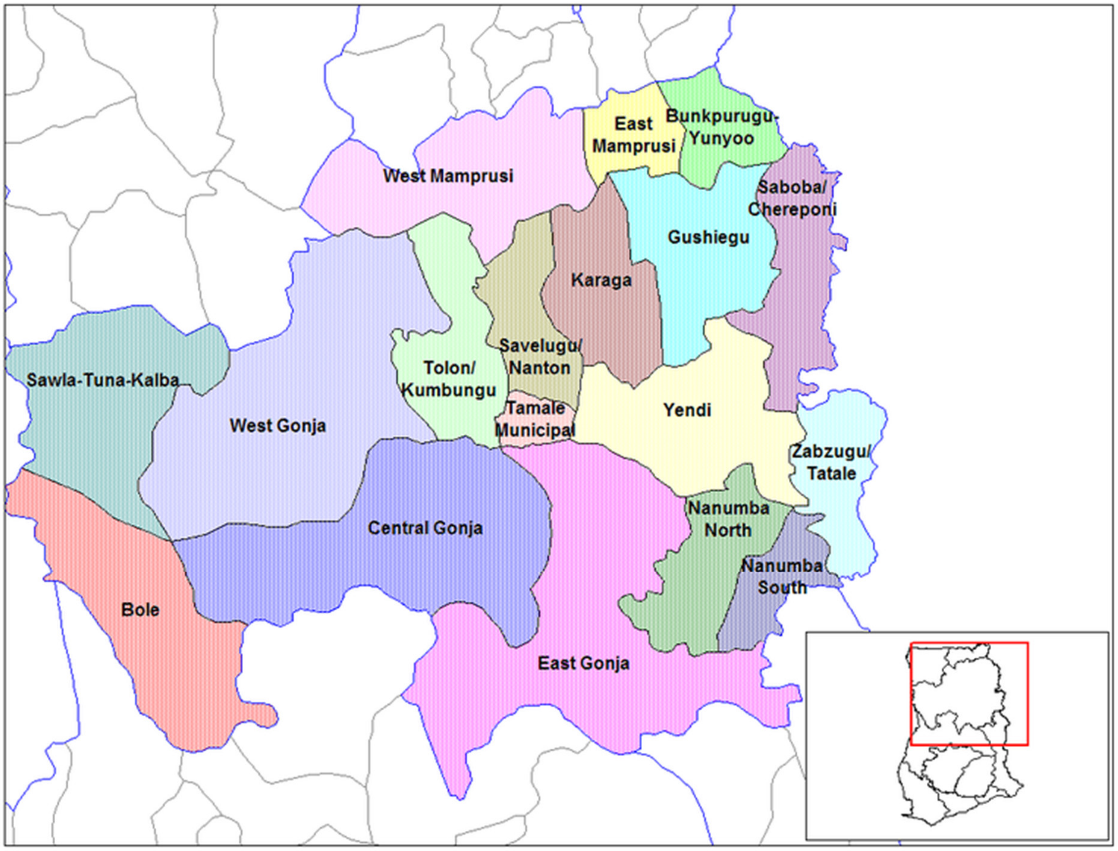

Figure S1. Illustrative Map of the Northern Region of Ghana (www.ghanaweb.)

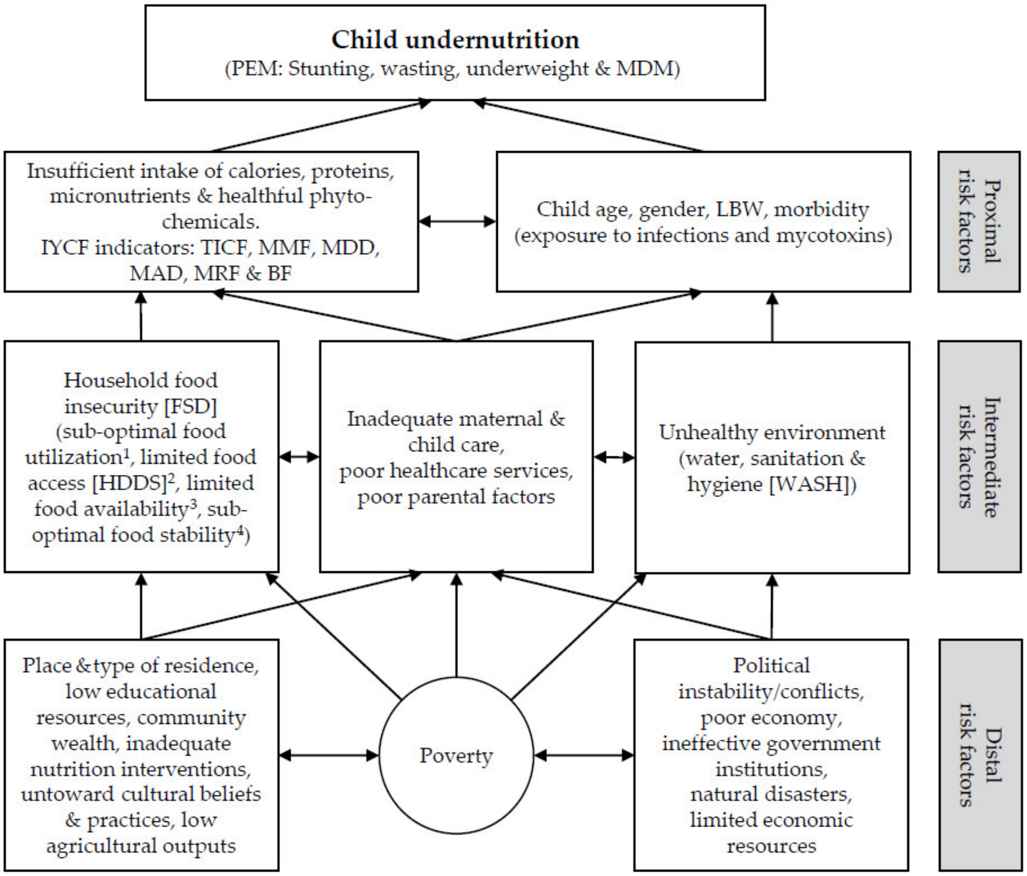

Figure S2. Conceptual Frame work for the Determinants of Malnutrition in Northern Ghana (Adopted and modified from Fanzo, 2012, Muller and Krawinkel, 2005 & UNICEF, 1998). [1] Sub-optimal food utilization: usage of nutritionally deleterious complementary food ingredient production practices such as Traditional Cereal Processing Methods (TCPMs), untoward Habitual Food Resource Utilization Practices (HFRUPs) such some traditional/indigenous food preparation methods, meal spoilage via lack of/poor storage facilities, post-harvest losses, poor knowledge of nutrition and untoward cultural beliefs and practices among other factors. [2] Limited food access (HDDS): low purchasing power, limited diversity of plant and animal food resources and inequitable household food /meal distribution [3] Limited food availability: inadequate food production, transportation and/or supply to the households' reach [4] Sub-optimal food stability: inconsistency in food production, supply, availability, access and utilization. FSD: Food Security Dimensions (Food Availability, Access, Utilization and Stability); HDDS: Household Dietary Diversity Score; IYCF: Infant and Young Child Feeding; LBW: Low Birth Weight; MAD: Minimum Acceptable Diet; MDD: Minimum Dietary Diversity; MDM: Micronutrient Deficiency Malnutrition; MMF: Minimum Meal Frequency; MRF: Micronutrient Rich Foods; BF: Breastfeeding; MRF: Micronutrient-Rich Foods; PEM: Protein Energy Malnutrition; TICF: Timely Introduction to Complementary Feeding.

Table S1. Socio-demographic and Household Characteristics *n* = 581.

Sociodemographic Characteristics	Frequency (*n*)	%
Communal Characteristics		
**District**		
Tamale	118	20.3
Karaga	125	21.5
Nanumba North	116	20.0
Tolon-Kumbungu	118	20.3
Central Gonja	104	17.9
**Religion**		
Islam	524	90.2
Christianity	36	6.2
Traditional African Religion (ATR)	21	3.6
**Ethnicity**		
Gonja	63	10.8
Dagomba	396	68.2
Nanumba	48	8.3
Kokomba	52	9.0
Others	22	3.8
**Residence/Community Type**		
Rural	421	72.5
Semi-rural	142	24.4
Urban	18	3.1
Household Characteristics		
**Type of Toilet Used**		
Improved	76	13.1
Not Improved	505	86.9
**Main Source of Drinking Water**		
Improved	303	52.2
Not Improved	278	47.8
**Main Source of Power for Lighting**		
Electricity	529	91
Others (Kerosene Lamp. Etc.)	52	9
**Fuel Used for Cooking**		
Modern (Electricity. LPG)	5	0.9
Others (Charcoal. Firewood)	576	99.1
**Household Wealth Index (HWI) or Socio-Economic Status (SES)**		
Low wealth (Poor)	232	39.9
Medium wealth (Average)	234	40.3
High wealth (Rich)	115	19.8
**Number of People Living in the Household**		
1–5 people	104	17.9
6–10 people	260	44.8
11 or more people	217	37.3
**Number of Rooms per Household**		
4 or Less	275	47.3
5–8	237	40.8
9 or More	69	11.9
**Number of People per Room**		
3 or Less	502	86.4
4 or more people	79	13.6

Table S2. Bivariate analysis of the association between factors and stunting (LAZ).

Factors		Nutritional Status (*n*)% Normal	Stunted	Total (*n*)%	Test Statistic	
**District of Residence**	Tamale	(79) 66.9	(39) 33.1	(118) 100.0	$\chi^2 = 31.874$	$p < 0.001$
	Tolon	(72) 61.0	(46) 39.0	(118) 100.0		
	Gonja Central	(75) 72.1	(29) 27.9	(104) 100.0		
	Nanumba North	(98) 84.5	(18) 15.5	(116) 100.0		
	Karaga	(65) 52.0	(60) 48.0	(125) 100.0		
**Maternal Age (Years)**	15–24	(107) 78.7	(29) 21.3	(136) 100.0	$\chi^2 = 12.089$	$p= 0.002$
	25–34	(208) 64.8	(113) 35.2	(321) 100.0		
	35–50	(74) 59.7	(50) 40.3	(124)100.0		
**Religion**	Islam	(340) 64.9	(184)35.1	(524) 100.0	$\chi^2 = 10.325$	$p= 0.006$
	Christianity	(31) 86.1	(5) 13.9	(36) 100.0		
	ATR	(18) 67.0	(3) 14.3	(21) 100.0		
**Tribe**	Gonja	(49) 77.8	(14) 22.2	(63) 100.0	$\chi^2 = 25.343$	$p < 0.001$
	Dagomba	(239) 60.4	(157) 39.6	(396) 100.0		
	Nanumba	(40) 83.3	(8) 16.7	(48) 100.0		
	Komkoba	(44) 84.6	(8) 15.4	(52) 100.0		
	Others	(17) 77.3	(5) 22.7	(22) 100.0		
**Child Age (Months)**	6–11	(203) 83.9	(39) 16.1	(242) 100.0	$\chi^2 = 62.131$	$p< 0.001$
	12–17	(114) 61.6	(71) 38.4	(185) 100.0		
	18–23	(72) 46.8	(82) 53.2	(154) 100.0		
**Maternal Height (cm)**	160 cm & Above	(204) 72.3	(78) 27.7	(282) 100.0	$\chi^2 = 7.187$	$p= 0.007$
	Below 160 cm	(185) 61.9	(114) 38.1	(299) 100.0		
**PNC**	< 4 Times	(82) 77.4	(24) 22.6	(106) 100.0	$\chi^2 = 6.344$	$p= 0.012$
	≥ 4 Times	(307) 64.6	(168) 35.4	(475) 100.0		
**ITN**	No	(35) 55.6	(28) 44.4	(63) 100.0	$\chi^2 = 4.149$	$p= 0.042$
	Yes	(354) 68.3	(164) 31.7	(518)100.0		
**Number of Occupants Per Household**	7 people or less	(153) 71.8	(60) 28.2	(213) 100.0	$\chi^2 = 3.616$	$p= 0.057^{**}$
	> 7 people	(236) 64.1	(132) 35.9	(368) 100.0		

Significance at $^{**}p < 0.10$ and $p < 0.05$.

Table S3. Bivariate analysis of the association between WHO IYCF indicators and Wasting.

IYCF Indicators		Nutritional Status (*n*)% Not Wasted	Wasted	Total (*n*)%	Test Statistic	
**TICF**	No	(167)28.74	(28)4.82	(195)33.56	$\chi^2 = 0.015$	$p= 0.904$
	Yes	(332)57.14	(54)9.29	(386)66.44		
**MMF**	No	(156)26.85	(22)3.79	(178)30.64	$\chi^2 = 0.651$	$p= 0.420$
	Yes	(343)59.04	(60)10.33	(403)69.36		
**MDD**	<4 foods	(246)42.34	(41)7.06	(287)49.40	$\chi^2 = 0.014$	$p= 0.906$
	≥ 4 foods	(253)43.55	(41)7.06	(294)50.60		
**MAD**	No	(309)53.18	(46)7.92	(355)61.10	$\chi^2 = 1.006$	$p= 0.316$
	Yes	(190)32.70	(36)6.20	(226)38.90		
**ACF**	No	(362)62.31	(55)9.47	(417)71.77	$\chi^2 = 1.041$	$p= 0.308$
	Yes	(137)23.58	(27)4.65	(164)28.23		

**Fe Intake**	No	(188)32.36	(26)4.48	(214)36.83	$\chi^2 = 1.078$	$p = 0.299$
	Yes	(311)53.53	(56)9.64	(367)63.17		
**Vitamin A**	No Intake	(211)36.32	(33)5.70	(244)42.00	$\chi^2 = 0.167$	$p = 0.920$
	Low intake	(278)47.85	(47)8.09	(325)55.94		
	High intake	(10)1.72	(2)0.34	(12)2.07		

No Significance at $p < 0.10$ and $p < 0.05$.

Table S4. Bivariate analysis of the association between factors and wasting.

Factors		Nutritional Status (*n*)% Normal	Wasted	Total (*n*)%	Test Statistic	
**Religion**	Islam	(459) 87.6	(65) 12.4	(524) 100.0	$\chi^2 = 17.529$	$p < 0.001^{**}$
	Christianity					
	ATR					
**Marital Status**	Single	(11)68.8	(5) 31.3	(16) 100.0	$\chi^2 = 3.986$	$p = 0.046^{**}$
	Married	(488) 86.4	(77) 13.6	(565) 100.0		
**Tribe**	Gonja	(57) 90.5	(6) 9.5	(63) 100.0	$\chi^2 = 15.996$	$p = 0.003^{**}$
	Dagomba	(341) 86.1	(55) 13.9	(396) 100.0		
	Nanumba	(44) 91.7	(4) 8.3	(48) 100.0		
	Komkoba	(36) 69.2	(16) 30.8	(52) 100.0		
	Others	(21) 95.5	(1) 4.5	(22) 100.0		
**Gender**	Female	(255) 91.1	(25) 8.9	(280) 100.0	$\chi^2 = 11.987$	$p = 0.001^{**}$
	Male	(244) 81.1	(57) 18.9	(301) 100		
**Child Age (Months)**	6–11	(214) 88.4	(28) 11.6	(242)	$\chi^2 = 15.265$	$p = 0.001^{**}$
	12–17	(144) 77.8	(41) 22.2	(185) 100.0		
	18–23	(141) 91.6	(13) 8.4	(154) 100.0		
**Maternal BMI**	Underweight	(53) 76.8	(16) 23.2	(69) 100.0	$\chi^2 = 9.336$	$p = 0.025^{**}$
	Normal	(359) 86.1	(58) 13.9	(417) 100.0		
	Overweight	(61) 88.4	(8) 11.6	(69) 100.0		
	Obese	(26) 100.0	(0) 0.0	(26) 100.0		
**Utility Power Source for Lighting**	Non-Electricity	(37) 71.2	(15) 28.8	(52) 100.0	$\chi^2 = 10.226$	$p = 0.001^{**}$
	Electricity	(462) 87.3	(67) 12.7	(529) 100.0		
**Child Morbidity (recent 2 weeks)**	No	(322) 88.0	(44) 12.0	(366) 100.0	$\chi^2 = 3.570$	$p = 0.059^{*}$
	Yes	(177) 82.3	(38) 17.7	(215) 100.0		
**Child Morbidity (Diarrhoea frequency in last 6 months)**	None	(163) 87.6	(23) 12.4	(186) 100.0	$\chi^2 = 6.272$	$p = 0.099^{*}$
	Only once	(171) 87.2	(25) 12.8	(196) 100.0		
	2–3 times	(146) 84.9	(26) 15.1	(172) 100.0		
	Every month	(19) 70.4	(8) 29.6	(27) 100.0		
**Child Immunization Status**	Not Up to Date	(171) 82.2	(37) 17.8	(208) 100.0	$\chi^2 = 3.610$	$p = 0.057^{*}$
	Up to Date	(328) 87.9	(45) 12.1	(373) 100.0		
**PNC**	< 4 Times	(85) 80.2	(21) 19.8	(106) 100.0	$\chi^2 = 3.472$	$p = 0.062^{*}$
	≥ 4 Times	(414) 87.2	(61) 12.8	(475) 100.0		
**Consumption of Cereal-Only-Based Meals**	Yes. Always	(119) 86.9	(18) 13.1	(137) 100.0	$\chi^2 = 6.519$	$p = 0.089^{*}$
	Yes. Very Often	(116) 89.9	(13) 10.1	(129) 100.0		
	Yes. Sometimes	(151) 80.7	(36) 19.3	(187) 100.0		
	No	(113) 88.3	(15) 11.7	(128) 100.0		

Significance at $^{*}p < 0.10$ and $^{**}p < 0.05$. ATR-African Traditional Religion BMI-Body Mass Index.
